# Factors associated with exclusive breast-feeding: A cross-sectional survey in Kaiyuan, Yunnan, Southwest China

**DOI:** 10.1371/journal.pone.0223251

**Published:** 2019-10-09

**Authors:** Yuan Ruan, Qiang Zhang, Juanjuan Li, Rong Wan, Jun Bai, Wenzhong Wang, Yutong Zhou, Qingqing Wan, Jiang Zhao, Siyang Yu, Min Peng, Zhitao Liu

**Affiliations:** 1 Department of Nutrition and Food Hygiene, Yunnan Centre for Disease Control and Prevention (CDC), Kunming, Yunnan, China; 2 Department of Public Health, Kaiyuan Centre for Disease Control and Prevention (CDC), Honghe, Yunnan, China; University of Virginia, UNITED STATES

## Abstract

Breastfeeding has a wide range of benefits for both infants and mothers. The identification of factors associated with exclusive breastfeeding (EBF) are important to increase the prevalence of EBF. The study aimed to determine the prevalence of EBF within the first six months and its associated factors in Kaiyuan, Yunnan Province, Southwest China. This cross-sectional study was conducted in Kaiyuan, a middle-sized city of Yunnan Province, Southwest China. Mothers of infants under twelve months were randomly selected for a face to face interview in four towns (two in urban areas and two in rural areas) in Kaiyuan. A structured questionnaire was applied for collection of sociodemographic information, mothers’ and infants’ health, and breastfeeding information. A 24-hour food recall survey was used to collect infant feeding information. A logistic regression analysis was performed to identify the factors independently associated with exclusive breastfeeding for infants up to six months of age. The number of 417 mothers with infants under six months was interviewed. The prevalence of EBF at six months was 27.34%. Logistic regression indicated that EBF within six months was more likely to be practiced by mothers who had higher average household income per year (OR = 2.09, 95% CI: 1.05–4.17 p = 0.037; OR = 1.85, 95% CI: 1.04–3.28 p = 0.037), and mothers who received breastfeeding information (OR = 2.46, (95%CI: 1.45–4.18, p = 0.0009). The prevalence of EBF in Kaiyuan, Southwest China is considerably lower than national and international recommendations. Yearly household income, and mothers who received breastfeeding information are associated with higher EBF prevalence. Breastfeeding information should be given to mothers in order to increase the prevalence of EBF.

## Introduction

Breastfeeding (BF) is a matter of concern in both developed and developing countries, because it has a wide range of benefits for infants as well as mothers. Breast milk is the first natural food for babies. It not only provides infants with the nutrients they need for health and development but also breastfeeding can make a significant reduction for all causes mortality and morbidity due to overall infections, such as gastrointestinal and respiratory tract infection [1–4]. The long-term benefits of breastfeeding for infants are also reported that it can promote cognitive development and increase educational attainment in adulthood [5,6], and protect infants against chronic diseases such as overweight, obesity, and diabetes in their later life [7]. Additionally, breastfeeding contributes to the health and well-being of mothers. It helps to increase the duration of lactational amenorrhoea and reduce the risk of ovarian carcinoma, breast cancer and type 2 diabetes mellitus [8,9].

Based on the benefits of breastfeeding, World Health Organization (WHO) recommended that all infants should be exclusively breastfed for the first six months of life since 2001[10]. Only 37% of infants younger than six months were exclusively breastfed in low-income and middle-income countries [11]. Although the prevalence of exclusive breastfeeding (EBF) increased slightly from 24.9% in 1993 to 35.7% in 2013 [11], this is far from the target as for 2025 to increase the prevalence of EBF in the first six months up to at least 50% set by the 56^th^ World Health Assembly [12]. In China, only 28.7% of infants within six months were exclusively breastfed in central and western China in 2010 [13]. This finding indicated that low prevalence of EBF is also the main challenge in China.

To increase the prevalence of EBF, it is vital to identify factors associated with EBF in order to do active support for establishing and sustaining appropriate breastfeeding practices. Some reviews have already reported that the determinants of breastfeeding is multi-dimensional, including socioeconomic, culture, social attitudes and values, legal and policy directives, woman’s work and employment conditions, health-care services and individual factors [14–16]. Although the factors associated with EBF and early initiation of breastfeeding have been broadly reported, studies are not sufficient in China. Therefore, the aim of the present study is to determine the role and value of some factors that may have positive or negative effects on the prevalence of EBF within six months.

## Materials and methods

### Subjects and study design

This is a cross-sectional study which was conducted in twelve counties in China during October and November 2017. First, the whole 31 province was divided into four strata, and probability proportionate to size sampling was used to select twelve counties. Kaiyuan with a population of 296,500 was selected as the middle-size cities to conduct this study. Then four towns (two in urban areas and two in rural areas) were selected by simple random sampling. Required number lists of eligible mothers with an infant less than twelve months of age were prepared in the selected areas with the help of local health facilities. Finally, 417 mothers who were healthy, could clearly answer questions and had an infant within six months from each area were randomly selected. The consent form was signed from all mothers before starting the interview. Ethical approval was obtained from National Institute of Nutrition and Health in Chinese Center for Disease Control and Prevention (CDC). Because we were provincial program group, we only had the right to analyze the data from Kaiyuan, Yunnan province, Southwest China.

### Sample size

The estimated sample size was calculated as follows:

$$\text{N}=\text{deff}\frac{u^{2}p(1-p)}{d^{2}}$$

d = r×p (r = 20.0%, p = 20.8%, the prevalence of EBF in 2013, a previous national study)

u = 1.96, deff = 2, the no response rate was estimated as 10.0%

N = 2×366÷90% = 813

A final sample size of 813 was needed for each survey site, which was divided into twelve groups (one month for each group). One group for this study was to be around 68. In this paper, we needed to analyze the prevalence of EBF and its associated factors within six months. So at least 408 mother-infant pairs were needed. Finally, 417 mothers with an infant under six months were recruited.

### Data collection and variable definition

Data was collected by trained, experienced local staffs from CDC of Kaiyuan, using a standardized questionnaire. The questionnaire includes various parts as following: sociodemographic information such as sex, age, education, occupation, family income, family size, birth weight, premature birth, infant’s feeding information within 24 hours such as breast milk, water, juice, tea, formula, mother’s and infants’ health such as the current birth, the type of delivery, the mothers’ diseases, and infants’ health issue after birth, breastfeeding support from hospital, family, community and work place were interviewed (S1 Table).

According to the WHO definitions, exclusive breastfeeding (EBF) was defined as the infant only receives breast milk including expressed breast milk or breast milk from a wet nurse) without any additional food or drink, (no water, no juice, no non-human milk, no solids and/or semi-solids), but except for oral rehydration solution (ORS), vitamins, minerals and medications in the form of drops or syrups up to age of six month [17,18]. As soon as the infants received anything else other than breast milk, even a teaspoon of water, he/she was excluded from the EBF category. We categorized education level as low (illiterate or primary school), medium (junior school), and high (senior school or having a university degree); occupation as unemployed (household work, agriculture work and self-supporting work), employed (trading, civil servant, labor and other salaried jobs). Income was measured by household income per capita in the last year. Birth weight was categorized as low (<2500g) and average or greater (>= 2500g). The method of delivery was divided into vaginal and caesarean.

### Statistical analysis

The statistical analysis was performed by SAS 9.3 (SAS Institute, Cary, NC, USA). Categorical variables (e.g. education, occupation) were presented as percentage. Binary logistic regression analysis was used to determine the factors associated with EBF during the first six months of infants by the odds ratio (OR), adjusted odds ratio (AOR) and 95% confidence interval (CI) for different independent variables. The dependent variable was exclusive breastfeeding or not. The independent variables were based on the indicators which were reported by previous reports and included in the present survey. Finally, we included age, education, occupation, income, family size, geographic area, birth weight, sex of infant, delivery mode, current birth, illness of mothers and their infants, antenatal visit and breastfeeding information as independent variable. The final model was constructed using factors that had an association with exclusive breastfeeding based on the univariate association. Two sided *p* valued less than 0.05 was considered statistically significant.

### Ethics statement

This study was approved by Institutional Review Board at the China Center for Disease Control and Prevention. Signed informed consent was obtained from all the participants before the survey.

## Results

### Sociodemographic information of participants

The sociodemographic information of the participants was displayed in Table 1. It showed that more than half (66.19%) of the 417 eligible mothers were between 20 and 29 years of age, and 44.36% of the participating mothers had finished junior school. In all, 78.18% of them were in unemployed, and 41.97% of the mothers had household incomes below 9,999 RMB per year. Almost all (94.00%) the infants in the study were born at normal weight.

**Table 1:** Sociodemographic information of study participants (N = 417).

	n	%
Maternal age (years)		
-19	41	9.83
20–29	276	66.19
30-	100	23.98
Mother’s education		
Low	115	27.58
Medium	185	44.36
High	117	28.06
Father’s education		
Low	99	23.74
Medium	201	48.20
High	117	28.06
Mother’s occupation		
Unemployed	326	78.18
Employed	91	21.82
Father’s occupation		
Unemployed	268	64.27
Employed	149	35.73
Average income per year (RMB)		
<=9,999	175	41.97
10,000–29,999	72	17.27
>=30,000	170	40.77
Family size		
<=4	106	25.42
5-	311	74.58
Residence		
Urban	208	49.88
Rural	209	50.12
Birth weight (g)		
<2500	25	6.00
>=2500	392	94.00
Sex of infant		
female	206	49.40
Male	211	50.60
Current birth		
First	195	46.76
Second	205	49.16
Third or more	17	4.08
Delivery mode		
Vaginal	306	73.38
Caesarean	111	26.62

## The prevalence of EBF

The prevalence of EBF was 27.34% at the first six months of infant. Within six months of age, 37.65% of infants had been fed by breast milk and water; 10.07% of the infants had received formula; 19.66% of them were fed by solid or semi-solid foods; only 4.80% of them had never been breast-fed, and 0.48% of them were fed by breast milk and fruits juice, tea or sugar water.

Reasons commonly given for discontinuing breastfeeding before six months were insufficient milk (30.00%), mother thought the child is old enough to be weaned (20.00%), infants’ unwillingness to suck (20.00%), the problems of mothers, such as illness, going to work (20.00%) and other reasons (10.00%).

## Factors associated with EBF

The results of the logistic regression of factors associated with EBF among 417 participating mothers with an infant under six months were described in Table 2. A univariate analysis found that eight factors had significant association with EBF: mothers’ age, mothers’ education, fathers’ education, fathers’ occupation, household average income per year, current birth, delivery mode and receiving breastfeeding information. When adjusted for other variables, the odds of six-month EBF increased 2.09 and 1.85 times for higher yearly income compared with the group of average income per year below 9,999 yuan (95% CI: 1.05–4.17, p = 0.035; 1.04–3.28, p = 0.037). Mothers who received breastfeeding information were 2.46 times (95%CI: 1.45–4.18, p = 0.0009) more likely to exclusively breastfeed to six months than women who did not receive information of breastfeeding.

**Table 2:** Factors associated with EBF within six months in Kaiyuan, Southwest China (N = 417).

	Total	n (%)	Crude OR (95%CI)	Adjusted OR (95%OR)
Mothers’ age (years)				
-19	41	13 (31.71)	1.00 (ref.)	1.00 (ref.)
20–29	276	86 (31.16)	0.98 (0.48–1.97)	1.41 (0.60–3.27)
30-	100	15 (15.00)	0.38 (0.16–0.90)*	0.43 (0.15–1.29)
Mother’s education				
Low	115	52 (45.22)	1.00 (ref.)	1.00 (ref.)
Medium	185	40 (21.62)	0.33 (0.20–0.56)***	0.49 (0.24–0.99)
High	117	22 (18.80)	0.28 (0.155–0.507)***	0.43 (0.17–1.11)
Father’s education				
Low	99	44 (44.44)	1.00 (ref.)	1.00 (ref.)
Medium	201	47 (23.38)	0.381 (0.228–0.638)***	0.54 (0.26–1.10)
High	117	23 (19.66)	0.306 (0.167–0.560)***	0.51 (0.20–1.84)
Mother’s occupation				
Unemployed	326	95 (29.14)	1.00 (ref.)	
Employed	91	19 (20.88)	0.64 (0.37–1.12)	
Father’s occupation				
Unemployed	268	82 (30.60)	1.00 (ref.)	1.00 (ref.)
Employed	149	32 (21.48)	0.62 (0.39–0.99)*	1.40 (0.67–2.89)
Average income per year (RMB)				
<=9,999	175	33 (18.86)	1.00 (ref.)	1.00 (ref.)
10,000–29,999	72	31 (43.06)	3.25 (1.78–5.93)***	2.09 (1.05–4.17)*
>=30,000	170	50 (29.41)	1.79 (1.09–2.96)*	1.85 (1.04–3.28)*
Family size				
<=4	106	25 (23.58)	1.00 (ref.)	
5-	311	89 (28.62)	1.30 (0.78–2.17)	
Residence				
Urban	208	62 (14.87)	1.00 (ref.)	
Rural	209	52 (12.47)	0.78 (0.51–1.20)	
Birth weight (g)				
<2500	25	6 (24.00)	1.00 (ref.)	
>=2500	392	108 (27.55)	0.83 (0.32–2.14)	
Sex of infant				
female	206	55 (26.70)	1.00 (ref.)	
Male	211	59 (27.96)	1.07 (0.69–1.64)	
Current birth				
First	195	56 (28.72)	1.00 (ref.)	1.00 (ref.)
Second	205	47 (22.93)	0.74 (0.47–1.16)	0.90 (0.52–1.56)
Third or more	17	11 (64.71)	4.55 (1.61–12.90)**	3.48 (0.98–12.36)
Delivery mode				
Vaginal	306	94 (30.72)	2.02 (1.174–3.47)	1.56 (0.84–2.88)
Caesarean	111	20 (18.02)	1.00 (ref.)	1.00 (ref.)
Mother suffers from chronic disease				
Yes	13	1 (7.69)	0.22 (0.03–1.67)	
No	404	113 (27.97)	1.00 (ref.)	
Infant suffers from disease after birth				
Yes	186	42 (22.58)	0.644 (0.41–1.00)	
No	231	72 (31.17)	1.00 (ref.)	
Antenatal care visits				
Yes	400	111 (27.75)	1.792 (0.51–6.36)	
No	17	3 (17.65)	1.00 (ref.)	
Received BF information				
Yes	233	78 (33.48)	2.07 (1.31–3.26)**	2.46 (1.45–4.18)***
No	184	36 (19.57)	1.00 (ref.)	1.00 (ref.)
Received free samples of breast milk substitutes				
Yes	8	2 (25.00)	0.88 (0.18–4.44)	
No	409	112 (27.38)	1.00 (ref.)	

n (%): the number and percentage of EBF for infants within six months divided by the independent variable

OR and 95% CI are adjusted for all other variables in the table

*P<0.05,

**P<0.01,

***P<0.001

## Results and discussion

This study identified the prevalence of EBF and its associated factors within six months in Kaiyuan, Yunnan Province, Southwest China. The Global Breastfeeding Scorecard found that only 40% of children younger than six months are breastfed exclusively in 194 nations, and China was included [19]. According to the data from the present survey, the prevalence of EBF was 27.34% within the first six months, which is comparable to previous surveys both nationally in 2008 and the 12 provinces of China in 2010 [13]. There were 37.65% of mothers fed their children breast milk and water. Actually, breast milk can provide all nutrients for the infants in first six months of life to achieve optimal growth, development and health. When children grew older, a large proportion of solid and/or semi-solid food was introduced to an infant. The WHO recommends that the introduction of complementary food should be nutritious, clean and safe, fed in adequate amounts, and not earlier than six month to keep young children healthy during this period. However, there was an ancient history of the early introduction of complementary feeding in China [13]. This traditional belief was influential in Kaiyuan with large minority ethnic populations. We found that 19.66% of infants younger than six months were fed by solid or semi-solid foods in this study. Only a few infants had never been fed by breast milk in our study, but mothers ceased breastfeeding earlier because of insufficient milk. Perceived insufficiency of milk was reported previously as a key factor leading to early breastfeeding cessation [20]. Actually, there were not many women who had physiological insufficient milk supply but most of them doubted their ability of breastfeeding result in the perception about lack of breast milk [21]. Decreasing mothers’ disbelief in her ability and increasing the self-efficacy to breastfeed is a strong support for continued breastfeeding [18,20,21]. Another most important reason for weaning was that mothers thought their children were old enough to be weaned in our study. This misconception leads to breastfeeding cessation earlier. Mothers should be educated that they need pay more attention for the benefits of breastfeeding not the age of weaning. Our findings showed that the prevalence of exclusive breastfeeding was still low compared to the global and national recommendation. Therefore, it is evident that there is room for improvement in infant feeding practices.

There are various contributing factors that may relate to the practice of exclusive breastfeeding [14]. With regard to socio-demographic characteristics, the maternal age and parental education were negatively correlated with EBF in univariate analysis of the present study. Maternal age was the factor most widely investigated, which was similar with ours that older mothers were less likely to EBF, BF and a weak intention to exclusively breastfeed an infant than younger ones [22–24]. However, other studies had reported that younger mothers were at a higher risk of early cessation of EBF [25]. Even though the older age effect on breastfeeding remains to be elucidated, it has to be taken into account that an increased maternal age at first childbirth has been recorded in most developed countries in the past 20 years. Hence, a high proportion of mothers aged 35 years or older may require specific attention [26,27]. A number of studies had observed that maternal and paternal educations were positively associated with EBF, BF duration and early initiation of BF [18,28–30]. In our study, mothers’ and fathers’ education were negatively associated with EBF in univariate analysis. However, the correlation disappeared after adjustment of covariates. Nearly seventy percent of participants completed the secondary school in the present study, while we found the majority of subjects finished academic colleges or universities studies in previous studies [18,30]. Thus, we recognized that the education was lower in the present study than previous studies. Mothers with lower education should be well-informed with the benefits of BF, and future breastfeeding programs should focus on maternal and paternal education status. High household income had a higher likelihood of EBF with multivariate logistic analysis in our study, which was also observed in a previous study [24]. This is because those mothers in the families with adequate amount of income do not need to go to work during lactation. Therefore, they have sufficient time to breastfeed their infants. In the view of maternal-child health, mothers with vaginal delivery had higher likelihood of EBF, although this relationship did not exist after adjustment of covariates. Similar findings were reported that caesarean delivery, the use of anaesthesia, maternal tiredness increase the risk of delaying of breastfeeding initiation as well as increase risk of prelacteal feeding [31,32]. Mothers who had third or more birth were at a higher probability of EBF. As a previous study revealed, and the multiparous women have higher self-confidence, self-efficacy, and infant feeding knowledge gained through earlier breastfeeding experiences and were more likely to breastfeed exclusively for six months [18,33]. Mothers who received information of improving BF were more likely to breastfeed exclusively to six months in our study. Similar findings were reported that breastfeeding knowledge, attitudes and interest were associated with higher odds of optimal breastfeeding practices [18,34]. The aforementioned determinants of BF such as age, education or household income are unable to take intervention, but the knowledge, attitudes/beliefs and practice of breastfeeding can be trained by health professionals, such as doctors, nurses, physical therapists and health care providers. The Baby Friendly Hospital Initiative (BFHI) by adopting the ten steps to successful breastfeeding was launched by the World Health Organization (WHO) and the United Nations Children’s Funds (UNICEF) in 1991. Breastfeeding initiation and the prevalence of EBF were increased among mothers with lower education who delivered in Baby-Friendly facilities [35]. We should focus on training health staff in skills to improve infant feeding practices especially in the economy depressing area, as they had frequently contact with pregnant women during prenatal care, pediatrician The findings have important implications for working to support new families in future of BF education, promotion and policy.

Admittedly, the study had the following limitation. We asked mothers to recall the infants feeding information at the past 24 hours. Recall bias could not be avoided as the information collected was based on the mother’s report of breastfeeding history. This recall bias might affect EBF prevalence in this cross-sectional study. Despite the limitation, this study has its strength. This is the first study to explore factors associated with EBF within six months in Kaiyuan, Southwest China. We identified that mothers who received information of breastfeeding associated with higher likelihood of EBF compared with those who did not received information of BF. Therefore, the results in this study give us a support for BF education, promotion and further studies in similar context.

### Conclusions

The findings of this study revealed that the prevalence of EBF at six months was 27.34% in Kaiyuan, Southwest China, which is considerably lower than national and international breastfeeding recommendations. Factors including yearly household income and mothers who received breastfeeding information were associated with higher prevalence of EBF in the present study. It is suggested the exclusive breastfeeding can be increased and promoted through breastfeeding education or training of mothers’ knowledge, awareness and practices.

## Supporting information

S1 Table Breastfeeding in Kaiyuan Southwest China anonymized. (XLS)
